# H_2_Mab-77 is a Sensitive and Specific Anti-HER2 Monoclonal Antibody Against Breast Cancer

**DOI:** 10.1089/mab.2017.0026

**Published:** 2017-08-01

**Authors:** Shunsuke Itai, Yuki Fujii, Mika K. Kaneko, Shinji Yamada, Takuro Nakamura, Miyuki Yanaka, Noriko Saidoh, Yao-Wen Chang, Saori Handa, Maki Takahashi, Hiroyoshi Suzuki, Hiroyuki Harada, Yukinari Kato

**Affiliations:** ^1^ Department of Antibody Drug Development, Tohoku University Graduate School of Medicine, Aoba-ku, Sendai, Miyagi, Japan.; ^2^ Department of Oral and Maxillofacial Surgery, Graduate School of Medical and Dental Sciences, Tokyo Medical and Dental University, Yushima, Bunkyo-ku, Tokyo, Japan.; ^3^ Department of Regional Innovation, Tohoku University Graduate School of Medicine, Aoba-ku, Sendai, Miyagi, Japan.; ^4^ Department of Pathology and Laboratory Medicine, Sendai Medical Center, Miyagino, Miyagino-ku, Sendai, Miyagi, Japan.; ^5^ New Industry Creation Hatchery Center, Tohoku University, Aoba-ku, Sendai, Miyagi, Japan.

**Keywords:** HER2, monoclonal antibody, immunohistochemistry, breast cancer

## Abstract

Human epidermal growth factor receptor 2 (HER2) plays a critical role in the progression of breast cancers, and HER2 overexpression is associated with poor clinical outcomes. Trastuzumab is an anti-HER2 humanized antibody that leads to significant survival benefits in patients with HER2-positive metastatic breast cancers. In this study, we developed novel anti-HER2 monoclonal antibodies (mAbs) and characterized their efficacy in flow cytometry, Western blot, and immunohistochemical analyses. Initially, we expressed the full length or ectodomain of HER2 in LN229 glioblastoma cells and then immunized mice with ectodomain of HER2 or LN229/HER2, and performed the first screening by enzyme-linked immunosorbent assays using ectodomain of HER2. Subsequently, we selected mAbs according to their efficacy in flow cytometry (second screening), Western blot (third screening), and immunohistochemical analyses (fourth screening). Among 100 mAb clones, only three mAbs reacted with HER2 in Western blot, and clone H_2_Mab-77 (IgG_1_, kappa) was selected. Finally, immunohistochemical analyses with H_2_Mab-77 showed sensitive and specific reactions against breast cancer cells, warranting the use of H_2_Mab-77 to detect HER2 in pathological analyses of breast cancers.

## Introduction

Human epidermal growth factor receptor 2 (HER2) plays an important role in the progression of breast cancers, and its overexpression was reported in >20% of patients with breast cancer.^(1,2)^ HER2 overexpression is associated with poor clinical outcomes, and the monoclonal antibodies (mAbs) trastuzumab^(3)^ and pertuzumab,^(4)^ the tyrosine kinase inhibitor lapatinib,^(5)^ and the antibody–drug conjugate (ADC) of trastuzumab and the maytansinoid microtubule assembly inhibitor trastuzumab emtansine^(6)^ have been approved for the treatment of HER2-positive breast cancers. Treatment with trastuzumab demonstrated significant survival benefits for patients with HER2-positive metastatic breast cancers.^(7)^ Furthermore, dual blockade of HER2 with the combination of pertuzumab and trastuzumab, as well as chemotherapy, led to significant improvements in overall survival compared with trastuzumab plus chemotherapy.^(8)^

Previously, we developed an original technology for the production of cancer-specific monoclonal antibodies (CasMabs) and anti-glycopeptide mAbs (GpMabs) against membrane proteins.^(9)^ We also developed sensitive and specific mAbs against several other membrane proteins using the same technology.^(10–13)^ In this study, we developed anti-HER2 mAbs for use in flow cytometry, Western blot, and immunohistochemical analyses.

## Materials and Methods

### Cell lines

LN229, A431, SK-BR-3, Chinese hamster ovary (CHO)-K1, HEK-293T, Met-5A, and P3U1 cell lines were obtained from the American Type Culture Collection (ATCC, Manassas, VA). LN229/HER2 and CHO/HER2 were produced by transfecting pCAG/PA-HER2-RAP-MAP^(14)^ into LN229 and CHO-K1 cells using Neon transfection system (Thermo Fisher Scientific, Inc., Waltham, MA) and Lipofectamine LTX (Thermo Fisher Scientific, Inc.), respectively. A few days after transfection, PA tag-positive cells^(15)^ were sorted using a cell sorter (SH800; Sony Corp., Tokyo, Japan).

CHO-K1, CHO/HER2, and P3U1 cell lines were cultured in RPMI 1640 medium (Nacalai Tesque, Inc., Kyoto, Japan), and LN229, LN229/HER2ec, LN229/HER2, A431, SK-BR-3, HEK-293T, and Met-5A cell lines were cultured in Dulbecco's modified Eagle's medium (Nacalai Tesque, Inc.), supplemented with 10% heat-inactivated fetal bovine serum (Thermo Fisher Scientific, Inc.), 100 units/mL of penicillin, 100 μg/mL of streptomycin, and 25 μg/mL of amphotericin B (Nacalai Tesque, Inc.) at 37°C in a humidified atmosphere containing 5% CO_2_ and 95% air.

### Animals

Female 4-week-old BALB/c mice were purchased from CLEA Japan (Tokyo, Japan). Animals were housed under pathogen-free conditions. The Animal Care and Use Committee of Tohoku University approved all of the animal experiments described herein.

### Tissues

One breast cancer patient who underwent surgery at Sendai Medical Center was recruited for examinations, and the ethics committee of Sendai Medical Center approved this study. Normal and cancer tissues were purchased from BioChain Institute, Inc. (Newark, CA) and US Biomax, Inc. (Rockville, MD), respectively.

### Hybridoma production

The ectodomain of HER2 with N-terminal PA tag^(15)^ and C-terminal RAP tag^(16)^ -MAP tag^(14)^ (HER2ec) was purified from supernatant of LN229/HER2ec using anti-MAP tag as described previously.^(14)^ BALB/c mice were immunized using intraperitoneal (i.p.) injections of LN229/HER2 cells or 100 μg of HER2ec together with Imject Alum (Thermo Fisher Scientific, Inc.). After several additional immunizations, a booster injection of LN229/HER2 cells or 100 μg of HER2ec was intraperitoneally administered 2 days before harvesting spleen cells. Spleen cells were then fused with P3U1 cells using PEG1500 (Roche Diagnostics, Indianapolis, IN) or GenomONE-CF (Ishihara Sangyo Kaisha, Ltd., Osaka, Japan).

The resulting hybridomas were grown in RPMI medium supplemented with hypoxanthine, aminopterin, and thymidine selection medium supplement (Thermo Fisher Scientific, Inc.). Culture supernatants were screened using enzyme-linked immunosorbent assay (ELISA) with HER2ec, and mAbs were screened using flow cytometry (second screening), Western blot (third screening), and immunohistochemical analyses (fourth screening). MAbs were purified from supernatants of hybridomas cultured in Hybridoma-SFM medium (Thermo Fisher Scientific, Inc.) using Protein G Sepharose 4 Fast Flow (GE Healthcare UK Ltd, Buckinghamshire, England).

### Flow cytometry

Cells were harvested by brief exposure to 0.25% trypsin/1-mM ethylenediaminetetraacetic acid (EDTA) (Nacalai Tesque, Inc.). After washing with 0.1% bovine serum albumin (BSA)/phosphate-buffered saline, the cells were treated with 1 μg/mL of anti-HER2 (clone H_2_Mab-77) for 30 minutes at 4°C and then with Oregon green-conjugated anti-mouse IgG (1:1000 diluted; Thermo Fisher Scientific, Inc.). Fluorescence data were collected using EC800 or SA3800 Cell Analyzers (Sony Corp.).

### Western blot analysis

Cell lysates (10 μg) were boiled in the sodium dodecyl sulfate (SDS) sample buffer (Nacalai Tesque, Inc.) and proteins were then electrophoresed on 5%–20% polyacrylamide gels (Wako Pure Chemical Industries Ltd., Osaka, Japan), and were transferred onto polyvinylidene difluoride (PVDF) membranes (Merck KGaA, Darmstadt, Germany). After blocking with 4% skim milk (Nacalai Tesque, Inc.), membranes were incubated with 1 μg/mL of primary antibodies, such as anti-HER2 (clone H_2_Mab-77) and anti-β-actin (clone AC-15; Sigma-Aldrich Corp., St. Louis, MO), and then with peroxidase-conjugated anti-mouse IgG (1:1000 diluted; Agilent Technologies, Inc., Santa Clara, CA), and were finally developed using ImmunoStar LD (Wako Pure Chemical Industries Ltd.) using a Sayaca-Imager (DRC Co. Ltd., Tokyo, Japan).

### Determination of the binding affinity using flow cytometry

A431 and SK-BR-3 (2 × 10^5^ cells) were suspended in 100 μL of serially diluted mAbs (0.6 ng/mL–10 μg/mL), and Alexa Fluor 488-conjugated anti-mouse IgG (1:1000; Cell Signaling Technology, Inc., Danvers, MA) was then added. Fluorescence data were collected using a cell analyzer (EC800; Sony Corp.). The dissociation constants (*K*_D_) were calculated by fitting the binding isotherms using the built-in one-site binding models in GraphPad PRISM 6 (GraphPad software, Inc., La Jolla, CA).

### Immunohistochemical analyses

Histologic sections of 4-μm thickness were deparaffinized in xylene and then rehydrated and autoclaved in citrate buffer (pH 6.0; Agilent Technologies, Inc.) for 20 minutes. Sections were then incubated with 1 μg/mL of primary mAbs for 1 hour at room temperature and then treated using an Envision+ kit (Agilent Technologies, Inc.) for 30 minutes. Color was developed using 3,3-diaminobenzidine tetrahydrochloride (Agilent Technologies, Inc.) for 2 minutes, and sections were then counterstained with hematoxylin (Wako Pure Chemical Industries Ltd.). The intensity of staining was evaluated as 0, 1+, 2+, and 3+.

## Results

### Production of anti-HER2 mAbs

Herein, we immunized mice with LN229/HER2 or purified recombinant HER2ec from culture supernatants of LN229/HER2ec cells. A booster i.p. injection of LN229/HER2 or HER2ec was also administered and culture supernatants were then screened for binding to purified HER2ec using ELISA. As a second screen, we used flow cytometry analyses to assess reactions with LN229 and LN229/HER2 cells. Because LN229 cells express endogenous HER2, a stronger reaction against LN229/HER2 was necessary.

A total of 29 clones were generated from immunizations with LN229/HER2, and 71 were generated from immunizations with purified HER2ec. Among 100 clones, 96 clones reacted with SK-BR-3 cells, which express endogenous HER2 (data not shown). Then, mAbs were further selected according to their efficacy on Western blot analysis. These analyses identified only one clone from LN229/HER2 immunizations and two clones from HER2ec immunizations that were useful for Western blot analyses, and the most sensitive clone H_2_Mab-77 (IgG_1_, kappa; from HER2ec immunization) was selected for subsequent studies.

### Characterization of H_2_Mab-77

**FIG. 1.**
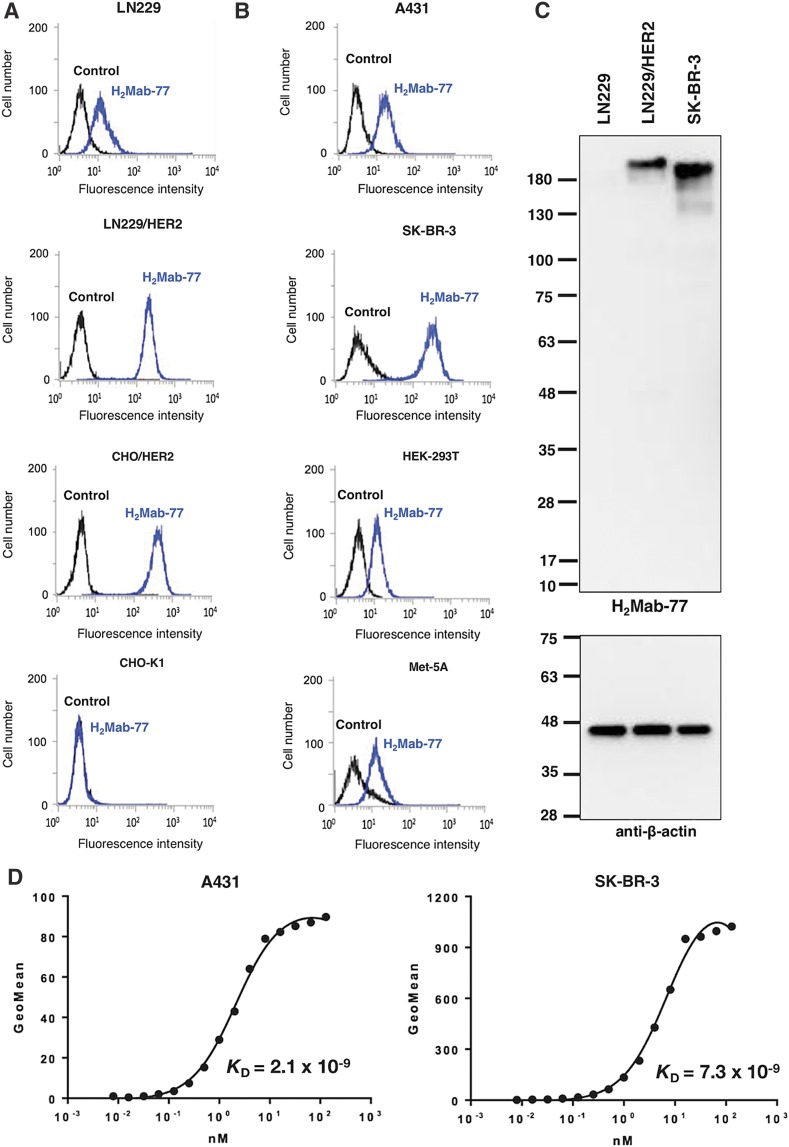
Characterization of H_2_Mab-77. **(A, B)** Flow cytometry with H_2_Mab-77; cells were treated with 1 μg/mL of H_2_Mab-77 followed by Oregon green-conjugated anti-mouse IgG; black line, negative control. **(C)** Western blot using H_2_Mab-77; cell lysates (10 μg) were electrophoresed and proteins were transferred onto PVDF membranes. After blocking, membranes were incubated with 1 μg/mL of H_2_Mab-77 or anti-β-actin (AC-15), and then incubated with peroxidase-conjugated anti-mouse IgG. **(D)** Binding affinity of H_2_Mab-77 was determined using flow cytometry. A431 and SK-BR-3 cells were suspended in 100 μL of serially diluted H_2_Mab-77 (0.6 ng/mL–10 μg/mL) and secondary anti-mouse IgG was then added. Fluorescence data were collected using a cell analyzer. PVDF, polyvinylidene difluoride.

In flow cytometric analyses, H_2_Mab-77 reacted with LN229/HER2 more strongly than with endogenous HER2-expressing LN229 glioblastoma cells (Fig. 1A). H_2_Mab-77 also reacted with CHO/HER2 and did not react with the parental cell strain CHO-K1, indicating that H_2_Mab-77 is specific for HER2 (Fig. 1A). H_2_Mab-77 also recognized endogenous HER2 in A431 epidermoid carcinoma cells, SK-BR-3 breast cancer cells, HEK-293T kidney epithelial cells, and Met-5A mesothelial cells (Fig. 1B). In subsequent Western blot analyses against LN229, LN229/HER2, and SK-BR-3 cells (Fig. 1C), H_2_Mab-77 detected a 180–200 kDa protein in LN229/HER2 and SK-BR-3 cells, indicating that H_2_Mab-77 is very useful for Western blot analyses. We further determined the binding affinity of H_2_Mab-77 for A431 and SK-BR-3 cells using flow cytometry (Fig. 1D) and calculated *K*_D_ values for H_2_Mab-77 of 2.1 × 10^−9^ M against A431 and 7.3 × 10^−9^ M against SK-BR-3, indicating that H_2_Mab-77 possesses high affinity, particularly for A431 cells.

### Immunohistochemical analysis against breast cancers

**FIG. 2.**
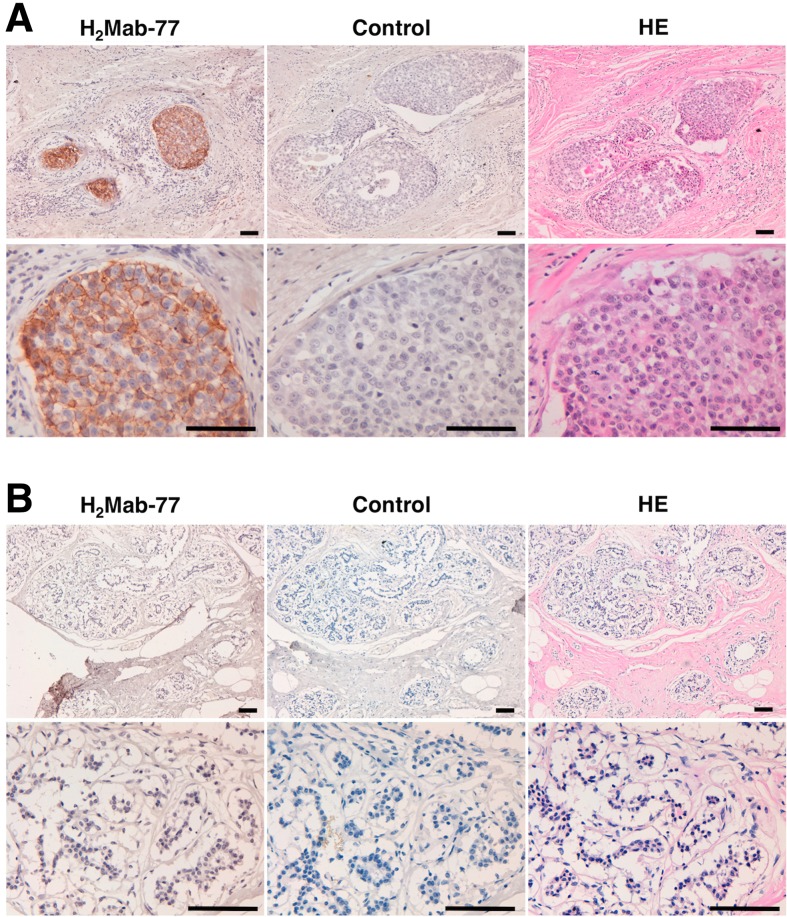
Immunohistochemical analysis by H_2_Mab-77 against breast cancers. Sections were incubated with 1 μg/mL of primary H_2_Mab-77 for 1 hour at room temperature followed by treatment with Envision+ kit for 30 minutes. Color was developed using 3, 3-diaminobenzidine tetrahydrochloride (DAB) for 2 minutes, and sections were then counterstained with hematoxylin. **(A)** Breast cancer; **(B)** normal breast tissues; scale bar = 100 μm.

Finally, we investigated the immunohistochemical utility of H_2_Mab-77 in human breast cancers. As shown in Figure 2A, H_2_Mab-77 stained membranes of cancer cells in this patient, who was previously diagnosed with HER2-positive breast cancer using HercepTest. In contrast, H_2_Mab-77 did not stain any adjacent breast tissues (Fig. 2B).

In immunohistochemical analyses using H_2_Mab-77 against two breast cancer tissue microarrays (Supplementary Table S1), H_2_Mab-77 stained 13 of 58 (22.4%) invasive ductal carcinomas and one medullary carcinoma (Supplementary Table S2). Typical staining patterns are presented in Supplementary Figure S1.

## Discussion

Establishment of mAbs that are useful for flow cytometry, Western blot, and immunohistochemical analyses is generally difficult. Moreover, designs of immunogens and screening methods to produce specific mAbs vary between Western blot and immunohistochemical applications, and it is very difficult to produce specific and sensitive mAbs that detect endogenous proteins. In this study, we used our original method for producing mAbs and immunized mice with LN229/HER2 or purified HER2ec from LN229/HER2ec cells. Among the 100 anti-HER2 mAbs developed herein, only three were applicable to Western blot analyses.

Among 100 clones of anti-HER2 mAbs, 97 (97%) were identified as belonging to the IgG_1_ subclass, and the remaining three to the IgG_2b_ subclass. H_2_Mab-77 was also determined to be of the IgG_1_ subclass, precluding confirmation of antibody-dependent cellular cytotoxicity (ADCC) or complement-dependent cytotoxicity (CDC) activities using H_2_Mab-77. Thus, in future studies, we will convert the subclass H_2_Mab-77 into mouse IgG_2a_ or IgG_2b_ subclasses or human IgG_1_, and assess their applications to measure ADCC/CDC activities.^(12,17,18)^

We previously produced an anti-podoplanin cancer-specific mAb (CasMab), clone LpMab-2^(9,18)^ or LpMab-23,^(19)^ which specifically recognizes cancer-type podoplanin in tumor tissues. For this technology, it is critical that immunogens are produced using cancer cell lines, such as LN229 glioblastoma cells, which express cancer-specific glycan-attached membrane proteins. This technology was also useful for generating anti-glycopeptide mAbs (GpMabs), including LpMab-3,^(20)^ LpMab-9,^(21)^ LpMab-12,^(22)^ LpMab-19,^(23)^ and LpMab-21.^(24,25)^ Furthermore, we used this technology to generate mAbs that bind to various novel epitopes of podoplanin, including LpMab-7,^(10–12)^ LpMab-10,^(26)^ LpMab-13,^(27)^ and LpMab-17.^(28)^ We have also developed sensitive and specific anti-podocalyxin mAbs using the same methods.^(13)^ Among anti-podocalyxin mAbs, clone PcMab-47 was reportedly useful in flow cytometry, Western blot, and immunohistochemical analyses, warranting consideration of PcMab-47 in investigations of podocalyxin expression and function in cancers and normal tissues. Moreover, this method could be used to develop useful mAbs against multiple membrane proteins.

Many other studies about antibody production and antibody engineering targeting HER2 have been performed. Luo et al. recently reported the glycoengineering of pertuzumab and its impact on the pharmacokinetic/pharmacodynamic properties.^(29)^ They showed that fucose was critical for ADCC activity by influencing the interaction between pertuzumab and FcγRIIIa, and removal of sialic acid using sialidase hydrolysis increased the ADCC and CDC activity of pertuzumab. Lopez-Albaitero et al. demonstrated that a novel HER2/CD3 bispecific antibody (HER2-BsAb) recruits and activates nonspecific circulating T cells, promoting T cell tumor infiltration and ablating HER2-positive tumors, even when these are resistant to standard HER2-targeted therapies.^(30)^ Menderes et al. reported that SYD985, a novel HER2-targeting ADC, is more potent than trastuzumab emtansine (T-DM1) against epithelial ovarian cancer.^(31)^ Sokolova et al. reported that immunotoxin 4D5scFv-PE40, which comprises 4D5scFv anti-HER2 antibody as a targeting module and fragment of *Pseudomonas* exotoxin A as an effector (toxic), showed significant antitumor effect on HER2-positive cancer cells *in vitro* and on HER2-positive xenograft models *in vivo.*^(32)^ In the same way with those studies, our established H_2_Mab-77 should be converted to ADC, immunotoxin, bispecific antibody with CD3, or glycoengineering mAbs to target effectively HER2-positive breast cancers.

In conclusion, from 100 mAb clones, H_2_Mab-77 was highly efficacious in Western blot analyses and produced strong staining in breast cancers. Hence, the mAb H_2_Mab-77 was useful in all the present experiments and will likely be a useful tool for the pathological identification of HER2 breast cancers.

## Supplementary Material

Supplemental data

Supplemental data

Supplemental data
