# Supplementary material for: H2Mab-77 is a Sensitive and Specific Anti-HER2 Monoclonal Antibody Against Breast Cancer
Source: Monoclon Antib Immunodiagn Immunother. 2017 Aug 1;36(4):143–8. doi: 10.1089/mab.2017.0026 (PMC6985780; doi:10.1089/mab.2017.0026)
Supplement: Supplemental data [file Supp_Fig1.pdf]

Supplementary Data

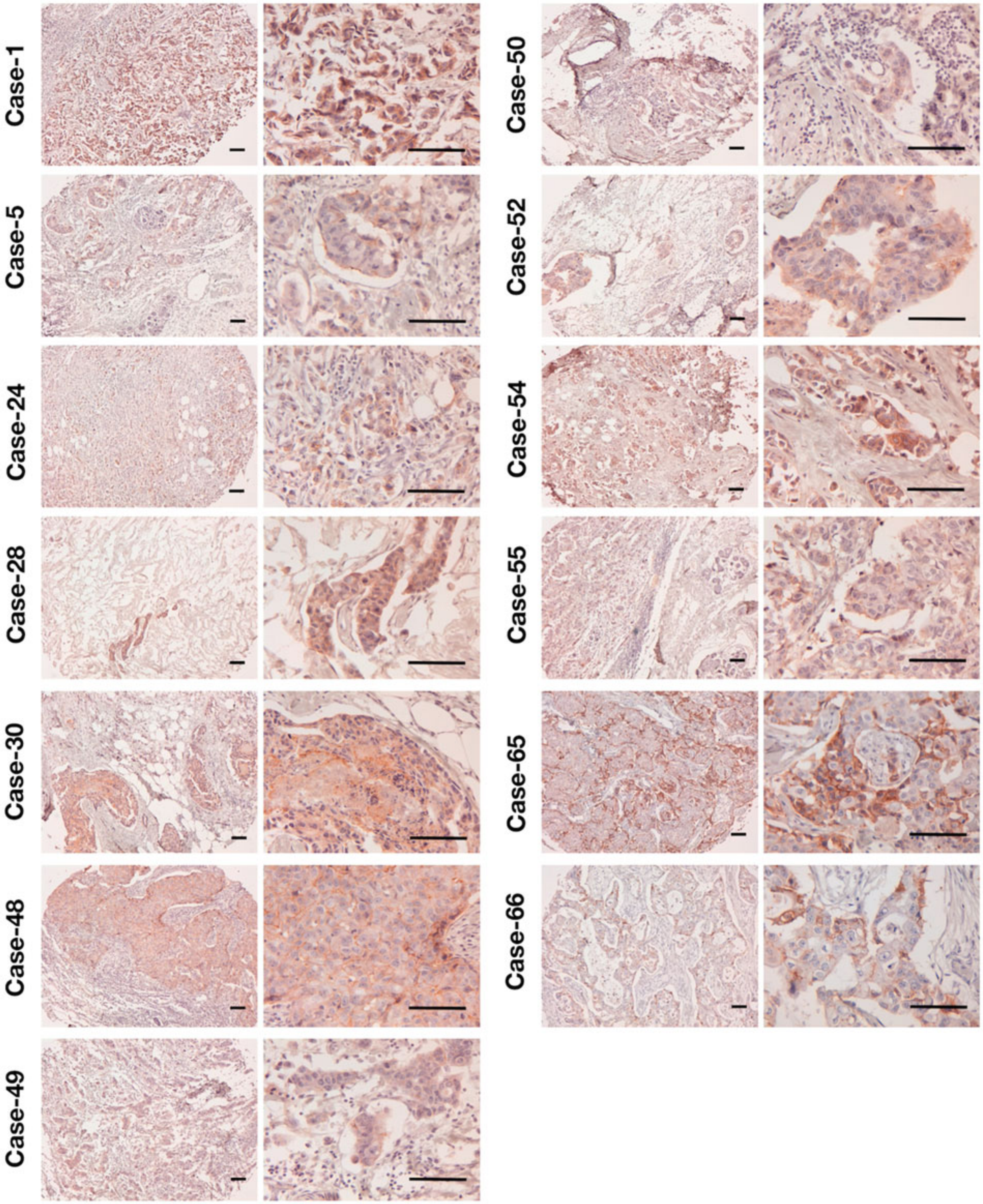

SUPPLEMENTARY FIG. S1. Immunohistochemical analysis by H_2Mab-77 against breast cancers. Sections were incubated with 1 μg/mL of H_2Mab-77 for 1 hour at room temperature followed by treatment with Envision+ kit for 30 minutes. Color was developed using 3, 3-diaminobenzidine tetrahydrochloride (DAB) for 2 minutes, and then the sections were counterstained with hematoxylin. Scale bar = 100 μm.
